# Influence of Composite Polishing Pastes on Surface Roughness and Their Stability After Simulated Tooth Brushing

**DOI:** 10.3390/dj13110528

**Published:** 2025-11-10

**Authors:** Panagiotis Ntovas, Bora Korkut, Nikolaos Loumprinis, Ioulianos Rachiotis, Christos Rahiotis

**Affiliations:** 1 Department of Operative Dentistry, Dental School, National and Kapodistrian University of Athens, Thivon 2, 11527 Athens, Greece; pntovas@dent.uoa.gr (P.N.); nikosloubr@hotmail.com (N.L.); 2 Department of Prosthodontics, School of Dental Medicine, Tufts University, Kneeland Street 1, Boston, MA 02111, USA; 3 Division of Fixed Prosthodontics and Biomaterials, University Clinics for Dental Medicine, University of Geneva, Rue Lombard 19, 1205 Geneva, Switzerland; 4 Department of Restorative Dentistry, Faculty of Dentistry, Marmara University, Istanbul 34854, Turkey; bora.korkut@marmara.edu.tr; 5 Dental School, National and Kapodistrian University of Athens, Thivon 2, 11527 Athens, Greece; sdn2100077@uoa.gr

**Keywords:** polishing paste, composite resin, surface roughness, tooth brushing, optical profilometer

## Abstract

**Background/Objectives:** Achieving and maintaining a smooth restoration surface is clinically significant, as surface roughness is linked to plaque accumulation, staining, and wear. This study aimed to evaluate in vitro the effect of different polishing paste systems on reducing surface roughness and to assess their performance after simulated post-operative maintenance through toothbrushing. **Methods:** A total of 128 cylindrical, flat-surface specimens were fabricated from a nanohybrid composite (Filtek Supreme XTE, 3M, USA) using a standardized metal mold. All specimens were finished with silicon carbide paper and polished with a two-step rubber disc system (Hi-Luster, Kerr, USA). They were then randomly assigned to 16 groups (*n* = 8) according to the polishing protocol. One group was polished with a prophylaxis paste, while the other fifteen groups were treated with pastes indicated for composite and/or ceramic materials. Polishing was performed with a flat buff wheel. To simulate clinical maintenance, specimens underwent a standardized toothbrushing cycle equivalent to three months of use. Surface roughness parameters (Sa and Sq) were measured at three stages with an optical profilometer: after initial polishing, after paste application, and after simulated toothbrushing. **Results:** Mean Sa values ranged from 0.065 to 0.560 and Sq values from 0.075 to 0.676. Significant differences were found among pastes for both parameters (*p* < 0.05). Two-way ANOVA revealed significant differences after polishing paste application, both before and after toothbrushing (*p* < 0.05). Toothbrushing increased roughness in most groups (*p* < 0.05), although no significant deterioration was observed for nine pastes in Sa and eight in Sq (*p* ≥ 0.05). **Conclusions:** Polishing pastes vary in effectiveness, and not all produce measurable improvements in surface smoothness. Their efficiency appears to be unrelated to the abrasive or the number of steps. Simulated toothbrushing over a three-month period may reduce the initial benefits, emphasizing the importance of careful clinical selection.

## 1. Introduction

Composite resins have been widely used in dentistry, either through direct or indirect techniques, including restorative procedures and temporary solutions in implant-supported restorations [1,2,3,4,5]. The clinical success and longevity of composite resins depend primarily on their surface characteristics, such as gloss and smoothness, as well as their ability to maintain these properties over time despite constant exposure to abrasives, such as tooth brushing [3,6].

Adequate surface smoothness of resin composites is of paramount importance, not only for aesthetic reasons but also for and gingival health, as it facilitates plaque control and preserves the marginal integrity of the restorative interface [4,5,7]. Furthermore, the surface roughness of composites can influence both their wear resistance and their potential to cause wear on opposing teeth [4,6].

Characterization of the surface texture of composite resins has become an essential factor in evaluating their clinical performance [5,6,8,9]. Surface analysis serves as a valuable diagnostic tool for comparing different types of composite resins and the effectiveness of various polishing protocols [8,9]. Surface roughness (Ra) can be assessed quantitatively using profilometry, as well as qualitatively through scanning electron microscopy (SEM) [10,11,12]. As Ra values increase, the risk of restoration discoloration or staining also rises, since rougher surfaces retain more pigments and reflect less light, thereby darkening the resin surface [11,12,13,14]. In addition, specific Ra thresholds have been proposed to enhance the clinical success of composite restorations [7,15,16,17,18]. For example, Ra values above 0.2 μm have been associated with an increased risk of plaque accumulation [7,15].

The surface roughness achievable in a composite restoration, as well as its retention during normal oral function and routine tooth brushing, depends on both the applied polishing protocol and the type of composite used [6,19,20,21]. Consequently, the best polishing technique to achieve and maintain the lowest possible surface roughness for each composite resin material has not yet been established, as different polishing systems can produce variable outcomes with different resin composites [14,17,22,23]. In particular, the chemical composition of a composite resin, along with mechanical characteristics determined by the size, shape, and proportion of inorganic filler particles, can significantly influence surface roughness [24,25,26,27,28,29]. Generally, smoother surfaces are obtained as filler particle size decreases and filler content by weight increases [19,20].

Regardless of the type of composite resin, finishing and polishing represent the final steps of a dental composite restoration, a provisional restoration, or an implant-supported individualized abutment, aiming to remove excess material while achieving the appropriate contour and anatomical form [2,3,8]. This is accomplished through a polishing protocol, which aims to reduce surface roughness and eliminate fine scratches produced during finishing, ultimately producing an optically and physically smooth surface [8,10,23]. In general, polishing methods are explained as a selective wear process that seeks to achieve a desirable surface texture through the sequential application of frictional agents with progressively smaller abrasive particle sizes. Traditionally, ideal polishing protocols employ a sequence of abrasive particles, starting with coarse grit and gradually decreasing in grit size [5,15].

A wide variety of one- and multi-step polishing systems have been introduced, including aluminum oxide-coated abrasive discs, abrasive-impregnated silicone or rubber cups, points, and discs, as well as abrasive strips and polishing pastes [11,24]. Most of these systems are coated or impregnated with aluminum oxide, silicon carbide, or diamond particles and are typically used in a predefined descending sequence of grain size [10]. Today, many polishing pastes are available on the market, offered as single-step or multi-step systems, with varying types, hardnesses, and sizes of abrasive particles [11,23].

The effect of polishing protocols on the roughness of different composite resins has been extensively investigated in vitro. Although the effect of polishing systems on composite surface roughness has been extensively investigated, the literature remains heterogeneous, with studies reporting conflicting results depending on the methodology, polishing sequence, and materials tested [11,13,25]. Such variability highlights the need for standardized comparative evaluations. In this context, the present study provides a comprehensive analysis of 16 different polishing pastes under identical experimental conditions, representing one of the broadest systematic comparisons reported to date and reinforcing the clinical relevance of our findings. The first null hypothesis was that no difference in surface roughness would be observed when a polishing paste is applied as an additional step after a two-step silicone polishing system, regardless of the type of polishing paste system used. The second null hypothesis was that simulated tooth brushing would result in similar surface roughness on the composite resin surface, irrespective of the previously applied polishing paste system.

## 2. Materials and Methods

### 2.1. Study Design

**Figure 1:**
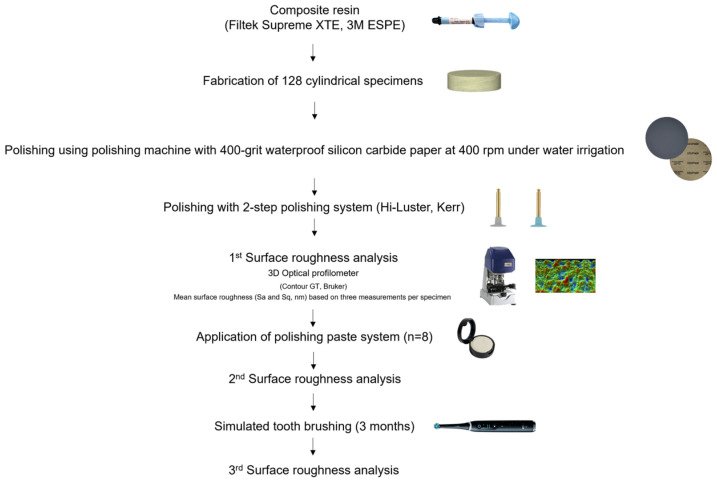
Flowchart of study’s design.

The present study was conducted at the National and Kapodistrian University of Athens, Greece. The study was performed in accordance with the CRIS guidelines for reporting in vitro studies [26]. The key components of the study’s design are presented through a flowchart in Figure 1.

### 2.2. Sample Size

Before initiating the study, a sample size estimation was performed using an online tool (G*Power, Ver. 3.1.9.7; Universität Kiel, Kiel, Schleswig-Holstein, Germany). Based on an estimated effect size of 0.7, an α error probability of 0.05, and a statistical power of 0.8, the required sample size was calculated to be 8 specimens per group [30,31,32]. As a result, a total of 128 specimens, each 8 mm in diameter and 3 mm in thickness, were fabricated using a nanohybrid composite material (Filtek Supreme XTE, 3M, St. Paul, MN, USA). The specifications of the evaluated composite resin are presented in Table 1.

### 2.3. Sample Fabrication

**Figure 2:**
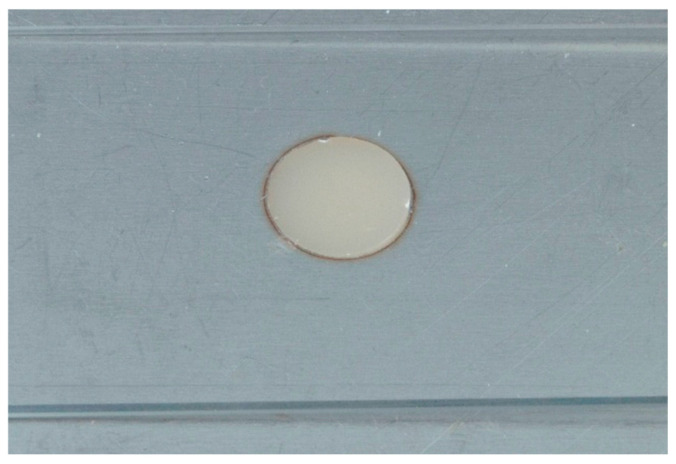
Fabrication of composite resin specimens using a laser-cut metal mold.

Disc-shaped specimens with flat surfaces were formed by placing the composite resin in a single increment into a metal mold made from an aluminum block, which was prepared using a fiber laser cutting machine (TruLaser Tube 5000, TRUMPF SE + Co. KG, Ditzingen, Germany). The free upper surface of the composite was covered with a Mylar strip and flattened with a glass slide (Figure 2).

Subsequently, the resin was polymerized using a polywave LED curing light (Valo X, Ultradent, Ultradent Products, Inc. South Jordan, UT, USA) according to the manufacturer’s instructions: 20 s from each side at an irradiation of 1400 mW/cm^2^. After the initial polymerization, each specimen was removed from the mold and light-cured for an additional 40 s. To ensure a uniform initial surface roughness, the specimens were finished using a waterproof abrasive silicon carbide paper of 400 grit on a wet polishing machine at a speed of 400 rpm. The specimens were then cleaned in an ultrasonic water bath for 2 min.

### 2.4. Initial Polishing

**Figure 3:**
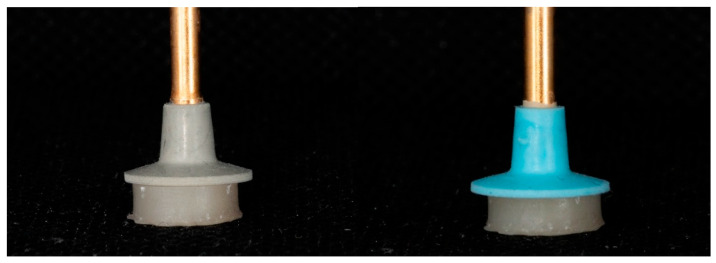
Polishing of specimens using a two-step system of rubber polishing discs.

After cleaning, each specimen was stabilized in a mold made of vinylpolysilicon (VPS) material. Subsequently, the specimens were polished using a two-step polishing system consisting of 2 rubber polishing discs (Hi-Luster, Kerr, Orange, CA, USA) (Figure 3).

**Figure 4:**
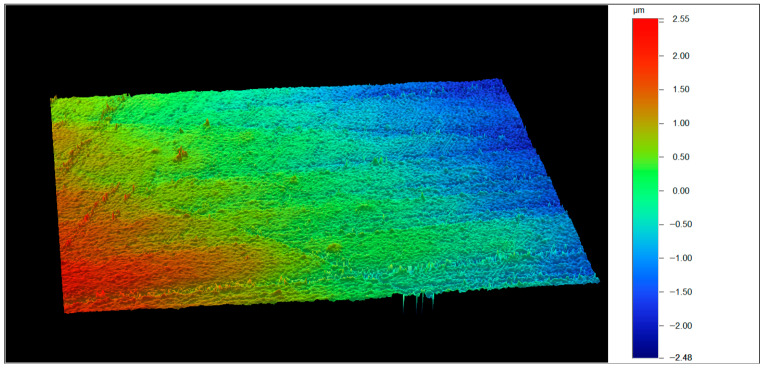
Measurement of specimen surface characteristics using 3D optical profilometer, 3D image of representative surface roughness.

An electric handpiece (W56, W&H) Dentalwerk Bürmoos GmbH, Bürmoos, Austria. was used to standardize the polishing speed at 8000 rpm. Each polishing disc’s flat surface was applied horizontally to the specimen surface 10 times for 2 s, and each disc was used only once per specimen. After polishing, the specimens were cleaned again in an ultrasonic water bath. The surface roughness of each specimen was assessed using vertical scanning interferometry (VSI) with a 3D optical profilometer (Contour GT, Bruker, Billerica, MA, USA) at 10× magnification. Data acquisition and calculation of the mean surface roughness in Surface Arithmetical mean Height (Sa), Sa units (nm), and Root Mean Square Height (Sq), units (nm) for each image were performed using the Vision64 (Bruker) software program (Figure 4).

A total of three images were captured for each specimen, and the values from these images were averaged to calculate the mean surface roughness for each specimen. An experienced operator performed all the assessments.

### 2.5. Additional Polishing

The composite resin specimens were randomly and equally divided into 16 groups of 8 specimens, according to the applied finishing and polishing protocol. One group was polished using a prophylaxis paste, while the remaining 15 groups were polished with pastes indicated for composite and/or ceramic materials. The specifications of the evaluated systems are presented in Table 2.

**Figure 5:**
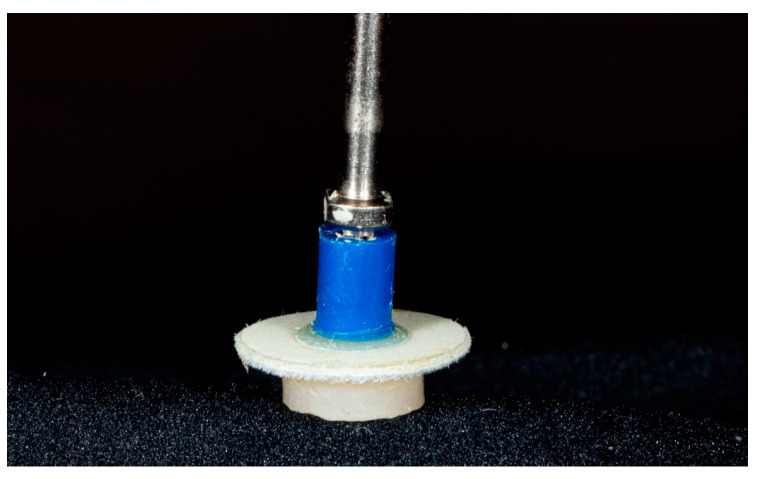
Polishing of specimens using flat buff wheels loaded with each evaluated paste system.

Each polishing paste was applied using a flat polishing pad buff wheel (Micro-cloth Polishing Disc, Shammy Bright, Kerr, Orange, CA, USA), with the pad contacting the specimen surface 10 times for 2 s per contact. Each polishing pad was used only once and then discarded (Figure 5).

Specimens were thoroughly rinsed with an air-water spray between each polishing step. After the final polishing, specimens were water-rinsed and placed in an ultrasonic bath for 2 min. To minimize variability, all specimen preparation, finishing, and polishing procedures were performed by the same operator, following the manufacturer’s instructions regarding speed, pressure, and use of water. To standardize pressure, each specimen, along with its mold, was placed over a precision scale. The surface roughness of each specimen was then measured a second time using the same 3D optical profilometer.

### 2.6. Simulated Tooth Brushing

**Figure 6:**
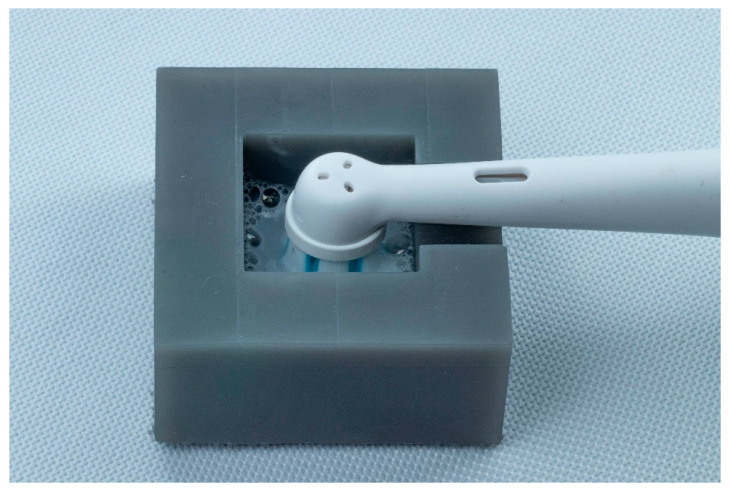
Brushing of the specimens using a toothbrush with an integrated pressure sensor.

Following the analysis, a simulated brushing cycle was performed on each specimen to mimic 3 months of clinical brushing [21,27]. According to ISO TR 1459-1:2007, all specimens were stored in water at 37 °C for 24 h before the brushing test [33]. Each specimen was fixed in a 3D-printed mold, which was filled with a dentifrice slurry made by mixing toothpaste (Total, Colgate, Colgate-Palmolive Company, NY, USA) and water in a 1:2 weight ratio. The mold was refilled to ensure complete coverage of the specimen during the brushing. An electric brush (iO, Series 7, Oral-B Procter & Gamble, Cincinnati, OH, USA), with a combination of pulsation and oscillation was attached to the mold. To standardize the applied pressure, the integrated smart sensor of the electric brush was used, indicating green light for favorable brushing pressure (0.8–2.5 N), red light for excessive force (>2.5 N), and white light for ineffective pressure (<0.8 N) (Figure 6) [28].

Based on an estimated maximum brushing time of 5 s per tooth surface per day, each specimen was brushed for 450 s to simulate 3 months of brushing [29]. After brushing, the specimens were rinsed with water and cleaned in an ultrasonic bath for 2 min. Their surface roughness was then measured for a third time using the same 3D optical profilometer.

To minimize operator-related variability throughout the experiment, each specimen was labeled with a unique number, and then a list of specimens was generated using a random sequence generator Random 2.7 (Random.org). This sequence was followed to assign polishing protocols randomly and to perform the brushing simulation on one specimen at a time before proceeding to the next.

### 2.7. Statistical Analysis

All statistical analyses were performed using IBM SPSS Statistics (version 25, IBM Corp., Armonk, NY, USA). The significance level was set at α = 0.05. The distribution of the data was assessed with the Shapiro–Wilk test and inspection of Q–Q plots. Homogeneity of variances at baseline was verified using Levene’s test. To evaluate comparability among groups after the initial two-step polishing, a one-way ANOVA was performed separately for Sa and Sq values. When significant, Tukey’s HSD post hoc tests were applied to identify pairwise differences among polishing groups. Because each specimen was measured at three stages (after initial polishing, after polishing paste application, and after simulated toothbrushing), a General Linear Model (GLM) with repeated measures ANOVA was employed to assess within-group changes over time. Mauchly’s test was used to examine the assumption of sphericity; when violated, the Greenhouse–Geisser correction was applied. Pairwise stage comparisons were conducted with Bonferroni-adjusted post hoc tests. Effect sizes were reported as partial eta squared (ηp^2^). Results are expressed as mean ± standard deviation (SD).

## 3. Results

The results of surface roughness for the composite specimens initially polished with the 2-step polishing system and subsequently polished with each of the investigated pastes, in terms of Sa and Sq parameters, are presented in Table 3.

The mean Sa values for the evaluated polishing paste systems ranged from 0.065 to 0.560, while Sq values ranged from 0.075 to 0.676. Two-way analysis of variance revealed significant differences among the evaluated specimens following the application of polishing pastes, both before and after simulated tooth brushing (*p* < 0.05).

Pairwise multiple comparisons showed that DiamondPolishingPaste (Strauss & Co), EnamePlusShiny (Micerium), PrismaGloss (Dentsply), DiamondPolish (Ultradent), Dura-PolishDia (Shofu), DiamontTwistSCO (Premier), Lucida (DiaShine), ZirconBrite (DVA), CompoSite (Shofu), CompoPlus (Fegupol), and Diabra (Fegupol) led significantly lower Sa values compared with the other investigated pastes (*p* < 0.05). Furthermore, EnamePlusShiny (Micerium), PrismaGloss (Dentsply), DiamondPolish (Ultradent), Dura-PolishDia (Shofu), DiamontTwistSCO (Premier), CompoSite (Shofu), CompoPlus (Fegupol), and Diabra (Fegupol) yielded significantly lower Sq values, compared to the rest of the investigated pastes (*p* < 0.05). Polishing with the systems mentioned above also resulted in significantly lower Sa and Sq values compared with specimens polished exclusively with the two-step polishing system (*p* < 0.05).

DiaPolisher (GC) and Speed Polish (Harvest Dental) polishing pastes, as well as Proxyt Fine (Ivoclar) prophylaxis paste, resulted in significantly higher Sa and Sq values compared to the other evaluated systems (*p* < 0.05). No significant differences were observed between initial and additional polishing when the same paste system was used (*p* ≥ 0.05).

**Figure 7:**
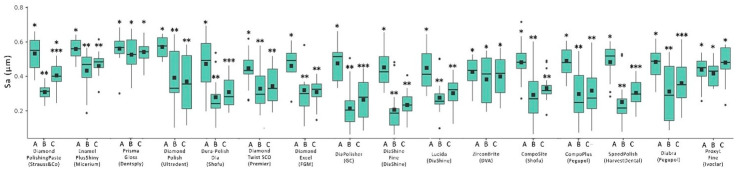
Boxplot of surface roughness (Sa) at 3 evaluation points: A: Two-step polishing system; B: Additional polishing with polishing paste; C: Simulated tooth brushing (3 months). Different symbols within each group indicate statistically significant differences among the three evaluation points (*p* < 0.05). Different asterisks within each polishing paste system indicate statistically significant differences among the three evaluation points (*p* < 0.05).

**Figure 8:**
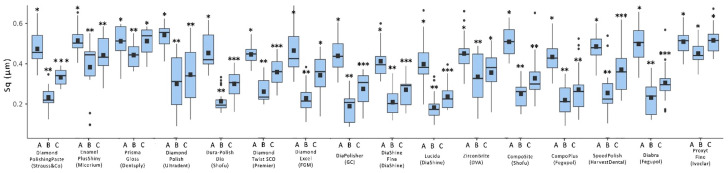
Boxplot of surface roughness (Sq) at three evaluation points: A: Two-step polishing system; B: Additional polishing with polishing paste; C: Simulated tooth brushing (3 months). Different symbols within each group indicate statistically significant differences among the three evaluation points (*p* < 0.05). Different asterisks within each polishing paste system indicate statistically significant differences among the three evaluation points (*p* < 0.05).

Simulated tooth brushing resulted in a significant increase in both Sa and Sq values for most of the evaluated pastes (*p* < 0.05). However, no significant differences were observed for 9 out of the 16 evaluated pastes with respect to Sa and for 8 out of the 16 with respect to Sq (*p* ≥ 0.05). Figure 7 and Figure 8 present boxplots of surface roughness for the Sa and Sq parameters, respectively, across the three evaluation points: initial polishing with a two-step polishing system, additional polishing with a paste, and simulated brushing (corresponding to a 3-month clinical period).

## 4. Discussion

The surface quality of resin composite restorations is a critical factor influencing their long-term clinical success, both esthetically and biologically. Inadequately polished restorations are associated with increased susceptibility to staining, plaque accumulation, gingival inflammation, and secondary caries. However, no standardized finishing and polishing protocol has yet been established for the different composite materials.

The present in vitro study aimed to evaluate the surface roughness and morphology of a nano-hybrid composite resin polished with a two-step rubber system, followed by the application of 15 different polishing pastes and 1 prophylaxis paste. Both surface roughness parameters, Sa and Sq, were analyzed in this study to provide complementary insights into the topography of the composite surfaces. Sa represents the arithmetical mean height of the surface deviations and reflects the overall smoothness. At the same time, Sq corresponds to the root mean square height, emphasizing the amplitude of peaks and valleys. Interpreting both parameters together offers a more comprehensive understanding of surface texture, as Sq is more sensitive to isolated irregularities that may not substantially affect Sa. The present results show that both parameters followed the same trend across polishing systems, confirming the consistency and robustness of the findings.

Based on the findings of this study, both null hypotheses were rejected, as the effects of additional polishing and simulated brushing varied significantly among the evaluated polishing paste systems. Most of the polishing pastes tested resulted in a significant reduction in surface roughness, as measured by parameters Sa and Sq, which is consistent with previous research. A previous study reported that the application of Lucida (Diashine) polishing paste improved the roughness of composite resins initially polished with Sof-Lex (3M) discs, Diacomp Plus Twist Polishing Spiral Wheels (Eve), Enhance/PoGo (Dentsply), or Kenda rubber systems. In another study, the use of Diamond Polish Paste (Ultradent) further improved the surface roughness of composites polished either with Sof-Lex (3M) discs or the Spiral (3M) rubber system [34]. The beneficial effect of using polishing paste systems has also been verified by other studies that tested various polishing pastes and materials [34,35,36,37,38,39,40]. In other studies, the application of polishing paste as an additional step did not lead to any further improvement in surface roughness and, in some cases, even had a detrimental effect [41,42,43,44,45]. However, other studies have reported that the effect of applying polishing pastes is influenced by both the type of composite resin material and the initial polishing method [13,46,47]. It is essential to emphasize that the type of resin composite may have a greater impact on surface roughness, color stability, and susceptibility to staining than the polishing technique itself [48]. This suggests that the final surface properties of a composite are likely more dependent on the combination of restorative material and finishing system used [19,20,49].

In the present study, the application of the third step of a prophylaxis paste, as well as some of the evaluated polishing paste systems, did not result in a significant change in surface roughness compared to that achieved with the initial two-step rubber polishing system. Even if some polishing pastes were not capable of significantly reducing roughness when used as an additional polishing step, their potential in clinical practice should not be overlooked. Polishing using pastes can access areas where discs or conventional silicone or rubber polishing systems cannot, as the shape of these instruments, combined with the location of the restoration, often limits their clinical applicability [49].

The type of abrasive particles varied both among and within the investigated polishing paste systems. Based on the results of the present study, polishing pastes containing different types of abrasive particles were able to achieve similar surface roughness. Specifically, polishing pastes that produced the lowest surface roughness contained solely diamond or aluminum oxide particles, or a combination of the two. However, these results cannot be generalized to other composite resins, as the effect of abrasive particles also depends on the type of fillers present in each resin. Additionally, previous studies have shown that small-sized, diamond-containing polishing systems can create smoother surfaces compared to aluminum oxide, due to their ability to polish both filler particles and the resin matrix [35,48,49,50,51].

In the present study, polishing pastes with three, two, or a single step were all able to achieve similar surface roughness. Consequently, the outcomes appeared to depend more on the individual characteristics of each system rather than the number of polishing steps. Multi-step polishing paste systems use progressively smaller particles at each stage to remove scratches from the previous polisher, aiming to achieve a highly polished surface. In contrast, one-step polishing systems utilize a combination of different particle sizes to abrade the fillers simultaneously and polish the resin matrix.

A direct comparison of the present study’s results with those of other studies is limited due to substantial differences in study design, particularly regarding the combinations of materials and polishing protocols employed. Factors such as the average size, volume, shape, distribution of filler particles, the quality of filler–matrix adhesion, the presence of voids, and the type of resin composite matrix can all significantly influence the effectiveness of each polishing technique on surface roughness [52]. For the composite resin investigated in the present study, a significantly greater interaction between the finishing and polishing techniques has been reported compared to other types of composite resins [53].

Furthermore, additional variables related to the operator and the abrasive instruments, such as the shape, the stiffness, and the flexibility of the instruments, the type, amount, and size of impregnated particles, and the presence of moisture, also vary among previously conducted studies [11]. In the present study, each polishing method was applied for 20 s; however, continued improvement has been reported for up to 30 s for each step of a three-step polishing system [22,54]. Prolonged use of specific polishing systems should be avoided, as the frictional heat generated can induce microcracks in the polymer matrix, leading to a rougher surface in hybrid composites [23]. Additionally, various studies have employed different finishing methods before polishing, including silicone carbide abrasive papers or diamond burs, which can contribute to variability in results [14,38,46,48]. These factors may explain the wide range of surface roughness values reported in previous studies.

In the present study, simulated toothbrushing over 3 months caused a significant increase in surface roughness in only some of the evaluated groups. In scientific literature, most studies have reported a significant difference in surface roughness parameters before and after simulated brushing [13,55,56,57]. Despite the increase in Ra following toothbrush abrasion, most commercial composites typically maintain surface roughness values below the 0.2 µm threshold for plaque accumulation [7,18]. In contrast, the outcomes of the present study showed that, after toothbrushing, the roughness of all evaluated groups exceeded this threshold. In some groups, values surpassed 0.3 µm, a level that may be perceptible to the patient’s tongue [58]. In the present study, toothpaste with a moderate-to-low relative dentin abrasiveness (RDA) value was used. It should therefore be noted that toothbrush abrasion tests using other types of toothpaste may yield different results [59]. Beyond brushing, several other factors may affect polish retention over time, including the presence of saliva, bacterial biodegradation, occlusal forces, temperature fluctuations, the abrasive potential of certain foods, and variations in oral pH [60,61,62,63,64,65,66]. Despite using an electric toothbrush equipped with a pressure sensor to maintain a standardized force range (0.8–2.5 N), it is challenging to achieve perfectly uniform pressure across the entire specimen surface, particularly in the transition zones between the center and the edges. This limitation is well acknowledged in literature and represents an inherent challenge in in vitro brushing simulations. In the present study, this variability was minimized by real-time feedback from the integrated sensor and by ensuring that a single operator conducted all brushing procedures under identical conditions. The polishing duration adopted in this study was based on manufacturer guidelines and corroborated by previous reports demonstrating adequate surface refinement without thermal stress [29]. Although extending polishing up to 30 s has been shown to reduce surface roughness in some studies further, excessive duration may lead to local overheating, microcrack formation, or resin matrix softening. Therefore, the selected time represented a balanced and standardized protocol, ensuring comparability with prior investigations while avoiding heat-related artifacts.

In the literature, Ra values above 0.2 μm have been associated with potential bacterial plaque retention [7,18]. Plaque accumulation has also been reported to be similar for surface roughness values ranging from 0.7 to 1.4 μm [64]. Mean roughness values between 0.25 and 0.50 μm are perceptible by the tongue, potentially leading to patient discomfort [66]. Regarding esthetic properties, Ra values below 1 μm are generally considered sufficient to provide an optically smooth surface [67]. In this study, all resin composites produced optically acceptable Ra values with all the tested polishing systems. However, some polishing systems failed to achieve a mean surface roughness below the clinically suggested threshold of 0.2 μm. It is worth noting that clinical evidence supporting these thresholds remains limited; therefore, they should be considered as rough guidelines [68]. The clinical outcomes suggest that, although composites may be roughened by toothbrushing and other abrasive events, their surface characteristics generally remain acceptable and are typically well tolerated by patients [69,70].

Various in vitro methods have been employed to evaluate the roughness of resin composites, including mechanical and 3D optical profilometry for quantitative analysis, as well as scanning electron microscopy (SEM) for qualitative assessment [14,22,56,63]. In the present study, an optical profilometer was used to measure specimen surface quality; however, most surface roughness data in the literature have been obtained using contact stylus profilometers. Optical profilometry provides 3D analysis, delivering both quantitative measurements and detailed visual representations [51,70]. In the present study, Sa and Sq surface parameters have been evaluated. However, future studies should also measure parameters such as the maximum depth of valleys (Sv), the maximum height of peaks (Sp), and their difference (Sz), to better differentiate materials with similar Ra or Sa values. Surface gloss was not assessed in this study, as the primary objective was to quantitatively evaluate surface topography through roughness parameters (Sa and Sq), which are closely associated with plaque accumulation, wear resistance, and optical appearance. Although gloss is a clinically relevant property directly influenced by polishing, its measurement was beyond the scope of the present investigation. Future studies should integrate gloss assessment alongside surface roughness to provide a more comprehensive understanding of the optical and functional effects of different polishing systems.

The results of the present study should be interpreted with caution and may not be generalizable to other composite resins, polishing instruments, or techniques. Only one nanohybrid composite resin (Filtek Supreme XTE) was evaluated in this study to maintain methodological consistency and minimize material-related variability. However, this choice inherently limits the extrapolation of the findings to other resin formulations, such as bulk-fill, micro-hybrid, or nanofilled composites, which differ in filler size, morphology, and resin matrix composition. These variations can significantly influence surface roughness and polish retention, and therefore, future studies should include comparative analyses among different composite categories to verify the generalizability of the present results. Another limitation of this study is the absence of qualitative microscopy techniques (e.g., Scanning Electron Microscopy or Atomic Force Microscopy), which could provide visual insights into surface morphology. While 3D optical profilometry allowed precise quantitative evaluation of roughness parameters (Sa and Sq), future studies should combine profilometric analysis with SEM or AFM imaging to achieve a more comprehensive surface characterization. Additionally, although the polishing procedures were performed by a single operator at controlled speeds and average pressure, using consistent movements parallel to the flat surface, it cannot be guaranteed that the same level of pressure was applied throughout the process. This potential variability may have introduced operator bias and influenced the study outcomes. In the present study, composite resin specimens with flat surfaces were used. Flat polishing buffs/discs have been implemented; however, in this configuration, the central part of the buff/disc moves in a different motion and at a different speed compared to the edges. The same limitation also applies to the simulated tooth-brushing process. Additionally, in clinical practice, restorations often exhibit convex or concave morphologies, which can impact the effectiveness of polishing methods. Therefore, clinical studies evaluating the impact of polishing quality on the longevity of composite resin restorations are required. Finally, only one toothpaste with moderate abrasiveness (Colgate Total; RDA ≈ 70) was used in this study to standardize brushing conditions. However, toothpaste abrasivity is known to directly affect the degree of surface wear and retention of polish. Therefore, results obtained under the current conditions may differ if higher- or lower-abrasivity dentifrices were used. Future research should compare toothpastes with varying RDA values to better understand how this factor influences the long-term preservation of polished composite surfaces.

## 5. Conclusions

Within the limitations of this in vitro study, the following conclusions can be drawn:

- Clinicians need to recognize that the effectiveness of polishing paste systems varies among composite resins, as some systems may not effectively enhance the surface smoothness of restorations.
- The efficiency of each paste does not appear to be related to the type of abrasive particles or the number of polishing steps.
- Simulated toothbrushing over 3 months diminishes the beneficial effects achieved by the additional application of some of the investigated polishing paste systems.
- Further research is needed to identify the optimal combination of polishing paste and composite resin.

## Figures and Tables

**Table 1:** Specifications of the tested composite resin.

Composite Resin	Manufacturer	Shade	Type	Organic Matrix *	Abrasive Particles and Particles Size	Filler (vol%, wt%)	Batch Number
Filtek Supreme XTE	3M ESPE	A3E	Nanofill 5–75 nm 0.6–1.4 μm	BisGMA, UDMA, TEGDMA, PEGDMA, BisEMA	Silica (20 nm), Zirconia (4–11 nm), zirconia-silica nanoclusters (0.6–20 μm)	59.5/78.5	N767724

* **Bis-GMA** (bisphenol A-glycidyl methacrylate), **UDMA** (urethane dimethacrylate), **TEGDMA** (triethylene glycol dimethacrylate), **PEGDMA** (polyethylene glycol dimethacrylate), and **Bis-EMA** (bisphenol A diglycidyl methacrylate ethoxylated).

**Table 2:** Specifications of tested finishing and polishing systems.

Polishing System	Manufacturer	Steps	Abrasive Particle	Particle Size
Hi-Luster	Kerr (Orange, CA, USA)	2	GlossPlus Aluminum oxide particle-integrated polishers HilusterPlus diamond particle-integrated polishers	GlossPlus 10 μm HilusterPlus 5 μm
Diamond Polishing Paste	Strauss & Co (Raanana, Israel)	3	Diamond	2 μm (F) 1 μm (SF) 0.5 μm (XXF)
Enamel Plus shiny	Micerium (Avegno, Italy)	3	Diamond (A,B) Aluminum oxide (C)	3 μm (A) 1 μm (B) 0.5 μm (C)
Prisma Gloss	Denstply (Charlotte, NC, USA)	2	Aluminum oxide	1 μm 0.1 μm
Diamond Polish	Ultradent (South Jordan, UT, USA)	2	Diamond	1 μm 0.5 μm
Dura-Polish DIA	Shofu (Kyoto, Japan)	2	Aluminum oxide Diamond (DIA)	<1 μm <1 μm (DIA)
Diamond Twist SCO	Premier (Plymouth Meeting, PA, USA)	1	Diamond	3 μm
Diamond Excel	FGM (Joinville, SC, Brazil)	1	Diamond	2–4 μm
DiaPolisher	GC (Tokyo, Japan)	1	Diamond	<10 μm
Diashine Fine	DiaShine (Burlington, WA, USA)	1	Diamond	<10 μm
Lucida	DiaShine	1	Sub-micron hybrid compound	1 μm
ZirconBrite	DVA (Corona, CA, USA)	1	Diamond	1 μm
CompoSite	Shofu	1	Aluminum oxide	<1 μm
CompoPlus	Fegupol (Buchen, Germany)	1	Diamond	1 μm
Speed polish	Harvest Dental (LLC, Irvine, CA, USA)	1	Diamond	N/A
Diabra	Fegupol	1	Diamond	N/A
Proxyt fine	Ivoclar (Schaan, Liechtenstein)	1	Pyrogenic silicic acid	N/A

***N/A:*** *Not available information.*

**Table 3:** Surface roughness values (Sa and Sq, µm; mean ± SD) following polishing and simulated tooth brushing.

Polishing System	Manufacturer	Polishing		Tooth Brushing Simulation	
		Sa	Sq	Sa	Sq
Hi-Luster	Kerr	0.46 ^a^ (±0.09)	0.53 ^a^ (±0.09)	0.49 ^a,b^ (±0.09)	0.53 ^a^ (±0.09)
Diamond Polishing Paste	Strauss & Co 15	0.24 ^b^ (±0.08)	0.34 ^b^ (±0.13)	0.30 ^c^ (±0.09)	0.42 ^b^ (±0.12)
Enamel Plus shiny	Micerium	0.20 ^b,c^ (±0.06)	0.26 ^b,c^ (±0.07)	0.27 ^c^ (±0.07)	0.32 ^c,c^ (±0.07)
Prisma Gloss	Denstply	0.22 ^b,c^ (±0.08)	0.30 ^b,c^ (±0.13)	0.28 ^c^ (±0.11)	0.37 ^b.c^ (±0.12)
Diamond Polish	Ultradent	0.17 ^b^ (±0.07)	0.21 ^c^ (±0.08)	0.26 ^c^ (±0.08)	0.32 ^c.d^ (±0.11)
Dura-Polish DIA	Shofu	0.21 ^b,c^ (±0.10)	0.26 ^b,c^ (±0.09)	0.33 ^c^ (±0.12)	0.34 ^b,c^ (±0.09)
Diamond Twist SCO	Premier	0.22 ^b,c^ (±0.07)	0.29 ^b,c^ (±0.07)	0.34 ^c^ (±0.10)	0.35 ^b,c,d^ (±0.06)
Diamond Excel	FGM	0.31 ^c^ (±0.14)	0.35 ^b^ (±0.171)	0.36 ^c^ (±0.14)	0.40 ^b,c^ (±0.15)
DiaPolisher	GC	0.39 ^a^ (±0.13)	0.48 ^a,d^ (±0.12)	0.43 ^a,b,d^ (±0.10)	0.51 ^a^ (±0.09)
Diashine Fine	DiaShine	0.34 ^c^ (±0.11)	0.42 ^d^ (±0.13)	0.36 ^c,d^ (±0.09)	0.46 ^a,b^ (±0.11)
Lucida	DiaShine	0.18 ^b^ (±0.05)	0.21 ^c^ (±0.04)	0.27 ^c^ (±0.04)	0.33 ^c,d^ (±0.08)
ZirconBrite	DVA	0.23 ^b,c^ (±0.06)	0.32 ^b^ (±0.10)	0.31 ^c^ (±0.10)	0.39 ^b,c^ (±0.07)
CompoSite	Shofu	0.21 ^b,c^ (±0.06)	0.26 ^b,c^ (±0.06)	0.26 ^c^ (±0.06)	0.36 ^b,c,d^ (±0.06)
CompoPlus	Fegupol	0.18 ^b,c^ (±0.05)	0.21 ^c^ (±0.07)	0.26 ^c^ (±0.07)	0.29 ^d^ (±0.08)
Speed Polish	Harvest Dental	0.44 ^a^ (±0.06)	0.51 ^a (^±0.09)	0.51 ^a^ (±0.07)	0.56 ^a^ (±0.06)
Diabra	Fegupol	0.18 ^b,c^ (±0.05)	0.22 ^c^ (±0.07)	0.27 ^c^ (±0.06)	0.29 ^d^ (±0.10)
Proxyt Fine	Ivoclar	0.42 ^a^ (±0.06)	0.48 ^a,d^ (±0.07)	0.46 ^a,b,d^ (±0.06)	0.59 ^a^ (±0.07)

*a,b,c,d Different lowercase letters within each row indicate statistically significant differences (p < 0.05).*

## Data Availability

The original contributions presented in this study are included in the article. Further inquiries can be directed to the corresponding author.
